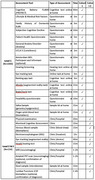# Prediction of Alzheimer's disease using an AI driven screening platform: interim results of the PREDICTOM study

**DOI:** 10.1002/alz70856_103775

**Published:** 2025-12-25

**Authors:** Anna‐Katharine Brem, Zunera Khan, Ellie Pickering, Jonas Botz, Johanna Mitterreiter, Nicholas J. Ashton, Martin Pszeida, Bin Huang, Sigurd Brandt, Richard Layton, Sarah Campill, Martha Therese Gjestsen, Paulina Tegethoff, Federica Cacciamani, Sara de Witte, Laura Ferré‐González, Augusto J. Mendes, Ana Bea Solana Sanchez, Spiros Nikolopoulos, Holger Fröhlich, Anne Corbett, Dag Aarsland, PREDICTOM Consortium

**Affiliations:** ^1^ University Hospital of Old Age Psychiatry, University of Bern, Bern, Switzerland; ^2^ King's College London, London, United Kingdom; ^3^ Institute of Psychiatry, Psychology and Neuroscience, King's College London, London, United Kingdom; ^4^ University of Exeter, Exeter, United Kingdom; ^5^ Fraunhofer SCAI, Sankt Augustin, NRW, Germany; ^6^ Siemens Healthineers AG, Forchheim, Germany; ^7^ Banner Sun Health Research Institute, Sun City, AZ, USA; ^8^ Banner Alzheimer's Institute and University of Arizona, Phoenix, AZ, USA; ^9^ JOANNEUM RESEARCH Forschungsgesellschaft mbH, Graz, Styria, Austria; ^10^ BrainCheck Inc., Austin, TX, USA; ^11^ GN brainworks, Ballerup, Denmark; ^12^ Muhdo, Ipswich, United Kingdom; ^13^ Alzheimer Europe, Luxembourg, Senningerberg, Luxembourg; ^14^ Centre for Age‐Related Medicine – SESAM, Stavanger University Hospital, Stavanger, Norway; ^15^ Department of Psychiatry and Psychotherapy, LMU Hospital, LMU Munich, Munich, Germany; ^16^ Qairnel, Paris, France; ^17^ Universitair Ziekenhuis Brussel, Brussel, Belgium; ^18^ Health Research INstitute La Fe, Valencia, Spain; ^19^ Geneva Memory Center, Department of Rehabilitation and Geriatrics, Geneva University Hospitals, Geneva, Geneva, Switzerland; ^20^ Laboratory of Neuroimaging of Aging (LANVIE), University of Geneva, Geneva, Geneva, Switzerland; ^21^ GE Healthcare, Munich, Bayern, Germany; ^22^ Centre for Research & Technology Hellas, Thessaloniki, Greece; ^23^ Fraunhofer Institute for Algorithms and Scientific Computing SCAI, Sankt Augustin, Germany; ^24^ King's College London, London, England, United Kingdom; ^25^ Centre for Age‐Related Medicine, Stavanger University Hospital, Stavanger, Stavanger, Norway

## Abstract

**Background:**

Early prediction of Alzheimer's disease (AD) is an urgent health challenge. The aim of PREDICTOM is to develop and test the accuracy of an artificial intelligence (AI) driven screening platform for the prediction and early detection of AD and to extend the clinical pathway to home‐based screening using established and novel biomarkers.

**Method:**

PREDICTOM is a pan‐European cohort study recruiting *N* = 4000 individuals over the age of 50 with increased risk of developing AD. Recruitment is expected to start in February 2025 across 7 European centres. Level 1 includes a home‐based assessment including digital (cognition, hearing, eye‐tracking, questionnaires) and physiological (finger‐prick blood, saliva) biomarkers. AI‐driven algorithms will be applied to identify participants with high (*N* = 415) versus low (*N* = 200) risk, who will enter the in‐clinic Level 2 including EEG, MRI, blood, cognition, hearing, stool and eye‐tracking measures (Table 1). The presence of AD pathology will be confirmed or ruled out using established biomarkers (cerebrospinal fluid, plasma, or amyloid PET) in Level 3. We will collect demographic, clinical and biomarker information and compare them across people with and without AD pathology using ANCOVA and hierarchical multiple regression (covariates: age, education, sex).

**Result:**

The interim analysis of Level 1 data will include N»1000 across the spectrum from high to low risk of AD.

**Conclusion:**

The PREDICTOM study will provide unique insights into diagnostic accuracy of at‐home measures for the early diagnosis of AD with initial results providing insights in their potential to differentiate between risk stages, specifically between the high and low risk stages. At AAIC, a full readout of the data from the first N»1000 participants from PREDICTOM will be presented.

**Acknowledgment**

This Project Is Supported By The Innovative Health Initiative Joint Undertaking (IHIJU) Under Grant Agreement No 101132356. The JU Receives Support From The European Union's Horizon Europe Research And Innovation Programme. This Work Was Funded By UK Research And Innovation (UKRI) Under The UK Government's Horizon Europe Funding Guarantee[UKRI Reference Number: 10083181]. In Switzerland The University Of Geneva Is Funded For PREDICTOM By The Swiss State Secretariat For Education Research And Innovation(SERI‐ Ref‐1131 52304).